# Supplementary material for: Asynchronous magnetic resonance elastography: Shear wave speed reconstruction using noise correlation of incoherent waves
Source: Magn Reson Med. 2022 Oct 27;89(3):990–1001. doi: 10.1002/mrm.29502 (PMC9792433; doi:10.1002/mrm.29502)
Supplement: Supplementary file 1 — DATA S1 MATLAB code used to generate the numerical simulations of Figure 1 [file MRM-89-990-s002.zip › k-Wave/helpfiles/hounsfield2density.html]

hounsfield2density :: Functions (k-Wave)


# hounsfield2density

Convert Hounsfield units to density.

## Syntax

```
density = hounsfield2density(ct_data)
density = hounsfield2density(ct_data, plot_fitting)
[density, sound_speed] = hounsfield2density(ct_data)
[density, sound_speed] = hounsfield2density(ct_data, plot_fitting)
```

## Description

`hounsfield2density` converts Hounsfield units to units of density [kg/m^3] based on the experimental data given by Schneider, U., Pedroni, E., and Lomax A., "The calibration of CT Hounsfield units for radiotherapy treatment planning," Phys. Med. Biol., 41, pp. 111-124 (1996). The conversion is made using a four-part piece-wise linear fit to the data (see figure below). For soft-tissue, the approximate sound speed can also be returned using the empirical relationship given by Mast, T. D., "Empirical relationships between acoustic parameters in human soft tissues," Acoust. Res. Lett. Online, 1(2), pp. 37-42 (2000).

## Inputs

|  |  |
| --- | --- |
| `ct_data` | CT data in Hounsfield units to convert to density |

## Optional Inputs

|  |  |
| --- | --- |
| `plot_fitting` | Boolean controlling whether the original data points and fitting is plotted (default = false) |

## Outputs

|  |  |
| --- | --- |
| `density` | density in kg/m^3 |
| `sound_speed` | sound speed in m/s |
